# Coronary Sinus Lead Entrapment During Transcatheter Tricuspid Valve Replacement: Implications for Pacing-Dependent Patients

**DOI:** 10.1016/j.jaccas.2026.108322

**Published:** 2026-05-14

**Authors:** Gabriel E. Soto, Shannon L. Tucker

**Affiliations:** Division of Cardiac Electrophysiology, Department of Internal Medicine, Missouri Baptist Medical Center, St Louis, Missouri, USA

**Keywords:** cardiac pacemaker, complication, tricuspid valve, valve replacement

## Abstract

**Background:**

Entrapment of transvenous pacing leads resulting in lead failure is a well-known complication of transcatheter tricuspid valve replacement (TTVR). Current guidelines advise preemptive alternative pacing strategies that do not cross the tricuspid valve in pacing-dependent patients.

**Case Summary:**

A pacing-dependent patient with a cardiac resynchronization therapy with defibrillator device underwent TTVR. He subsequently developed simultaneous fractures of his right ventricular and left ventricular leads, necessitating an epicardial pacing system.

**Discussion:**

Transvenous left ventricular pacing leads coursing near the tricuspid valve annulus can also be at risk of entrapment during TTVR, resulting in sudden lead failure.

**Take-Home Messages:**

Coronary sinus lead entrapment is a potential complication of TTVR, especially for leads coursing near the tricuspid valve annulus and/or when a large-diameter valve is deployed. TTVR in pacing-dependent patients in whom a pre-existing coronary sinus lead is the sole or backup means of pacing require meticulous attention during valve placement and close follow-up.

## Visual Summary

**Figure:**
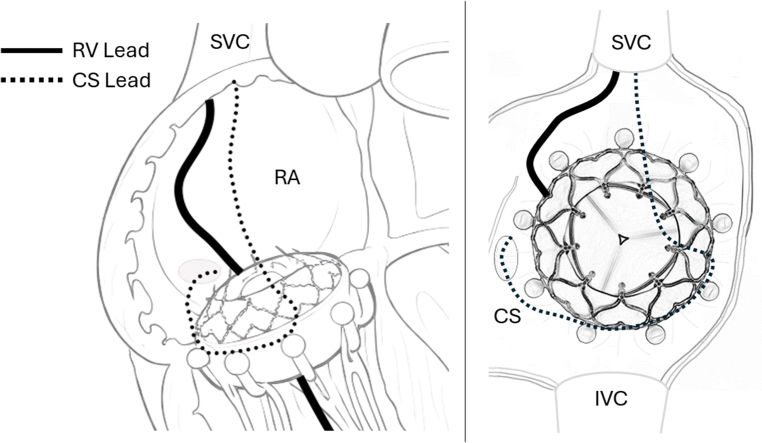

## History of Presentation

A 72-year-old man with a history of a nonischemic cardiomyopathy and cardiac resynchronization therapy with a defibrillator implant presented with progressive right upper quadrant abdominal pain and a tender palpable liver over the preceding several weeks.

**Visual Summary:**
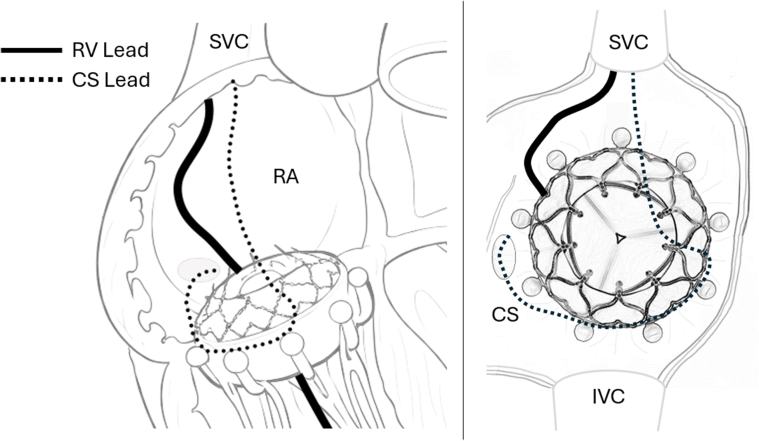
Impingement of RV and CS leads by TTVR Lateral (left) and en face (right) views of dual entrapment of RV and CS pacing leads by the EVOQUE valve. CS = coronary sinus; IVC = inferior vena cava; RA = right atrium; RV = right ventricular; SVC = superior vena cava.

**Take-Home Messages**

• Coronary sinus lead entrapment is a potential complication of TTVR, especially for leads coursing near the tricuspid valve annulus and/or when a large-diameter valve is deployed.
• TTVR in pacing-dependent patients in whom a pre-existing coronary sinus lead is the sole or backup means of pacing require meticulous attention during valve placement and close follow-up.

## Past Medical History

The patient had end-stage renal disease secondary to diabetic nephropathy for which he was on dialysis. He had a distant history of percutaneous coronary intervention to his left anterior descending coronary artery and had undergone implantation of a cardiac resynchronization therapy with defibrillator device in the setting of a severely depressed left ventricular ejection fraction (LVEF) of 25% with left bundle branch block; his LVEF subsequently improved to 55%. He went on to develop rapidly conducted paroxysmal atrial fibrillation refractory to attempts at pharmacologic management and ultimately underwent an ablation of his atrioventricular node to establish complete heart block. Additional comorbidities include Parkinson's disease, type 2 diabetes mellitus, and orthostatic hypotension requiring chronic midodrine therapy. He had no history of documented ventricular tachyarrhythmias.

## Investigations

His clinical history and examination were suspicious for cardiogenic cirrhosis. A right upper quadrant ultrasound confirmed diffusely increased hepatic echogenicity and nodularity consistent with hepatic cirrhosis as well as the presence of moderate ascites. Transthoracic and transesophageal echocardiography showed normal left ventricular (LV) systolic function (LVEF of 55%-60%), right ventricular (RV) enlargement with mild hypokinesis, and severe tricuspid insufficiency. A right and left cardiac catheterization showed no more than mild coronary artery disease, with a significantly elevated left ventricular end-diastolic pressure of 30 mm Hg and a pulmonary artery pressure of 70/41 mm Hg, respectively.

## Management

After aggressive attempts at medical optimization, a repeat right heart catheterization demonstrated an interval improvement of his pulmonary artery pressure to 45/16 mm Hg with a pulmonary capillary wedge pressure of 16 mm Hg. Repeat echocardiography showed persistent severe tricuspid insufficiency and an anatomy unsuitable for transcatheter edge-to-edge repair. His predicted operative mortality and combined morbidity and mortality for an isolated tricuspid valve repair were 6.36% and 18.4%, respectively, based on his Society of Thoracic Surgeons score. His Kansas City Cardiomyopathy Questionnaire overall summary score was 32. A decision was made to move forward with a palliative transcatheter tricuspid valve replacement (TTVR).

Electrophysiology was consulted regarding device and lead management options. A shared decision was made to forgo a transvenous lead extraction in favor of close postoperative surveillance of lead performance, with asynchronous LV pacing serving as a fallback in the event that noise reversion mode was triggered by an RV lead fracture. The patient subsequently underwent a TTVR with an EVOQUE 56-mm valve (Edwards Lifesciences) without acute complications, achieving a good echocardiographic result in terms of minimal residual tricuspid regurgitation. Intraoperative and predischarge interrogations of the cardiac implantable electronic device (CIED) showed no evidence of lead dysfunction.

## Outcome and Follow-Up

**Figure 1:**
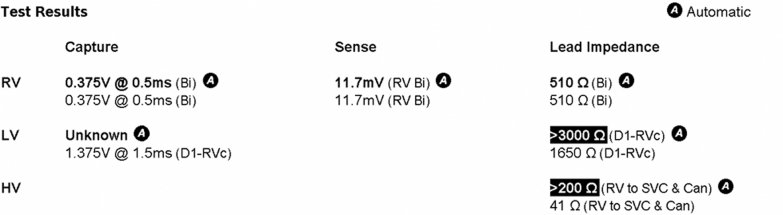
CIED Interrogation Screen capture of device interrogation at the 2-week follow-up demonstrating out-of-range impedance readings for LV lead and high-voltage coil on the RV lead as compared with previous measurements performed immediately after EVOQUE implant. HV = high voltage; LV = left ventricular; RV = right ventricular.

**Figure 2:**
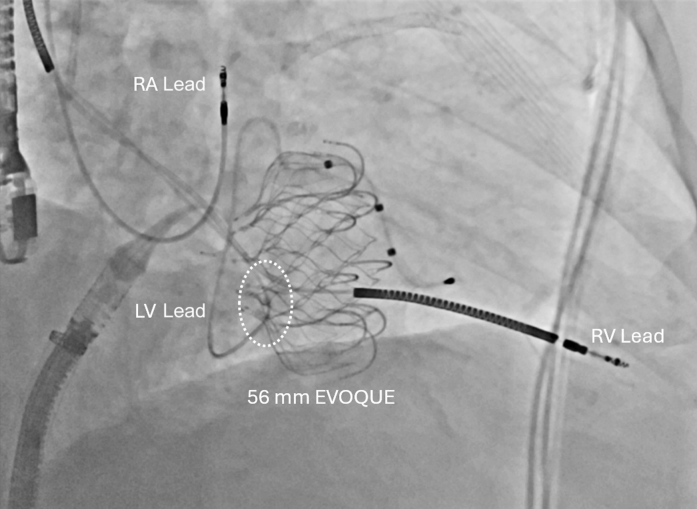
Fluoroscopic Image of TTVR Implant Fluoroscopic right anterior oblique projection after EVOQUE valve deployment showing impingement of the coronary sinus lead by the inferolateral edge of the valve (dotted circle), along with jailing of the RV lead. RA = right atrial; Abbreviations as in Figure 1.

On postoperative day 12, a high-voltage impedance alert was received via remote monitoring. The patient was seen in the device clinic the following morning in which an interrogation of his device demonstrated out-of-range impedances on both the high-voltage coil and the LV lead (Figure 1), with LV noncapture at high output in all pacing vectors consistent with dual fractures of both the RV coil and LV lead. Review of his intraoperative fluoroscopy images obtained during TTVR revealed a region of probable impingement of the LV lead as it traversed the right atrium along the edge of the tricuspid valve annulus before entering the coronary sinus (Figure 2).

The patient was directly admitted to the hospital and ultimately underwent placement of epicardial RV and LV pacing leads with a decision to forego antitachycardia therapies. He was discharged to home but had several subsequent admissions related to his cirrhosis and recurrent ascites. He ultimately developed *Staphylococcus aureus* bacteremia and died after transitioning to compassionate care approximately 4 months after his TTVR.

## Discussion

The optimal management of patients with transvenous CIED systems undergoing TTVR is an area of active debate, given the risks of RV lead damage resulting from entrapment during deployment of the prosthetic valve. Early reports suggest that the rates of lead failure are at least 10% to 20% within the first 12 months, often without any preceding warning indicators.^1^^,^^2^

Current consensus statements and guidelines recommend that pacemaker-dependent patients with transvenous leads crossing the tricuspid valve annulus who are scheduled for TTVR should have an alternative pacing modality, such as a leadless pacemaker, epicardial pacemaker, or coronary sinus lead, placed before TTVR to avoid the risks associated with transvalvular lead entrapment. The European Heart Rhythm Association and collaborating societies specifically advise preemptive alternative pacing strategies that do not cross the tricuspid valve and recommend avoiding transvenous RV leads during TTVR.^2^^,^^3^ Furthermore, new proposed guidelines from the Heart Rhythm Society advocate for extraction of any transvenous leads crossing the tricuspid valve annulus before TTVR, giving this a Class 1 recommendation based on expert opinion.^4^

The case presented herein highlights the complexities inherent to the management of these patients. To our knowledge, this is the first reported case of co-entrapment of an LV lead during TTVR deployment resulting in LV lead failure. This has implications for dependent patients who have an existing coronary sinus LV lead in place at the time of TTVR and suggests that routine reliance on the presence of a coronary sinus lead as a backup pacing modality may be neither safe nor reliable, especially with larger diameter prosthetic tricuspid valves.

A consideration during preprocedural planning for this patient was whether or not to extract the RV lead before proceeding with TTVR and leaving him with a left ventricle–only pacing system vs reimplanting an implantable cardioverter-defibrillator, subcutaneous implantable cardioverter-defibrillator, or extravascular implantable cardioverter-defibrillator. At our institution, we have adopted a multidisciplinary approach that addresses the following factors to help guide shared decision making: 1) estimated survival with TTVR; 2) estimated complication rates associated with jailed transvenous leads (aside from lead fractures); 3) estimated risks associated with prophylactic lead extraction and survivability of complications; and 4) long-term device needs.

Although long-term survival data with TTVR are currently lacking, the TRI-SCORE provides estimates for 2-year survival based on data from multicenter registry studies and cohort analyses.^5^ This patient had a TRI-SCORE of 7, with an estimated 2-year estimated survival rate of 68% and an extrapolated 5-year survival rate likely under 60%. Conversely, the risk of a CIED-associated infection over a 5-year period was estimated at 20% to 25% in this dialysis-dependent patient, with recognition that successful treatment of CIED-associated infections requires complete removal of all hardware, as multiple studies have demonstrated that the risk of infection relapse without complete hardware removal is high (between 50% and 100%).^6^^,^^7^

With respect to risks of extraction, periprocedural mortality with contemporary lead extraction techniques at experienced centers is relatively low at 0.3% to 0.5% in multicenter analyses, including the prospective European Lead Extraction Controlled Registry^8^; however, certain risk factors are known to increase the risk of perforation and other major complications such as pneumothorax and/or hemothorax, stroke, and pulmonary embolism. The CLEAR score, derived from the multicenter CLEAR (Canadian Lead ExtrAction Risk) study, has been shown to have reasonable discrimination for predicting the risk of perforation within 30 days.^9^ Similarly, the SAFeTY score has been validated for predicting the risk of any major complication associated with a lead extraction.^10^

For this patient, both his CLEAR and SAFeTY scores predicted periprocedural complication rates ≤0.5%. Ultimately, the patient opted to forgo a transvenous lead extraction in part because he was considered a poor surgical candidate in the event of a significant vascular complication and his desire to limit further procedural interventions pending his clinical response to TTVR. In hindsight, had he opted to undergo an RV lead extraction with preservation of his LV lead only, he likely would have experienced a lethal bradyarrhythmia when his LV lead failed without preceding warning indicators.

## Conclusions

Optimal transvenous lead management in patients undergoing TTVR remains an area of active investigation and debate, with pacing-dependent patients representing a group at particularly high risk of complications related to lead dysfunction. Although the presence of a coronary sinus LV pacing lead is generally considered an adequate backup pacing modality, this case demonstrates the possibility that even these can become entrapped during TTVR deployment, requiring close vigilance both intraoperatively and during post-TTVR follow-up.

## Funding Support and Author Disclosures

The authors have reported that they have no relationships relevant to the contents of this paper to disclose.
